# Factors Related to Tooth Loss Among Community-Dwelling Middle-aged and Elderly Japanese Men

**DOI:** 10.2188/jea.JE20120180

**Published:** 2013-07-05

**Authors:** Ayumi Ando, Masaki Ohsawa, Yumi Yaegashi, Kiyomi Sakata, Kozo Tanno, Toshiyuki Onoda, Kazuyoshi Itai, Fumitaka Tanaka, Shinji Makita, Shinichi Omama, Kuniaki Ogasawara, Akira Ogawa, Yasuhiro Ishibashi, Toru Kuribayashi, Tomiko Koyama, Akira Okayama

**Affiliations:** 1 Department of Hygiene and Preventive Medicine, School of Medicine, Iwate Medical University, Yahaba, Iwate, Japan; 1 岩手医科大学医学部衛生学公衆衛生学講座; 2 The First Institute of Health Service, Japan Anti-Tuberculosis Association, Tokyo, Japan; 2 結核予防会第一健康相談所生活習慣病予防研究センター; 3 Division of Cardiology, Nephrology and Endocrinology, Department of Internal Medicine, Iwate Medical University, Morioka, Japan; 3 岩手医科大学医学部内科学講座心血管・腎・内分泌内科分野; 4 Department of Neurosurgery, School of Medicine, Iwate Medical University, Morioka, Japan; 4 岩手医科大学医学部脳神経外科学講座; 5 Division of Neurology and Gerontology, Department of Internal Medicine, School of Medicine, Iwate Medical University, Morioka, Japan; 5 岩手医科大学医学部内科学講座神経内科・老年科分野; 6 Department of Health and Physical Education, Faculty of Education, Iwate University, Morioka, Japan; 6 岩手大学教育学部; 7 Iwate Health Service Association, Morioka, Japan; 7 岩手県予防医学協会

**Keywords:** tooth loss, risk indicator, middle-aged men, elderly men, Japanese, cross-sectional study

## Abstract

**Background:**

Using data from a large-scale community-based Japanese population, we attempted to identify factors associated with tooth loss in middle-aged and elderly men.

**Methods:**

A total of 8352 men aged 40 to 79 years who lived in the north of the main island of Japan and underwent health checkups were enrolled between 2002 and 2005. Number of teeth was assessed by the question, “How many teeth do you have (0, 1–9, 10–19, or ≥20)?”. On the basis of the answer to this question, participants were classified into 2 groups (≤19 teeth or ≥20 teeth). Using multivariate logistic regression, factors related to having 19 or fewer teeth were estimated.

**Results:**

The numbers (percentages) of participants who had 0, 1 to 9, 10 to 19, and 20 or more teeth were 1764 (21.1%), 1779 (21.3%), 1836 (22.0%), and 2973 (35.6%), respectively. Among the participants overall and those aged 65 to 79 years, having 19 or fewer teeth was significantly associated with older age, smoking status (current smoking and ex-smoking), and low education level. In addition, men with 19 or fewer teeth were more likely to have a low body mass index and low serum albumin level and less likely to be current alcohol drinkers. Among men aged 40 to 64 years, but not men aged 65 to 79 years, those with 19 or fewer teeth were more likely to have a low serum high-density lipoprotein cholesterol level and high glycosylated hemoglobin (HbA1c) level.

**Conclusions:**

Smoking, low education level, and poor nutritional status were associated with tooth loss among middle-aged and elderly Japanese men.

## Abstract (ja)

**背景目的:**

大規模な日本人の地域住民のデータを使用し、中高年男性の歯牙喪失に関連する要因を明らかにすること。

**方法:**

2002年-2005年に市町村による基本健診を受診し、研究参加に同意した東北地域（岩手県北部地域）に住む40-79歳の男性8352名を対象とした。現在歯数は単一の質問（あなたは何本の歯を持っていますか？（0本、1-9本、10-19本、20本以上））によって評価された。対象者はその質問の答えによって2つのグループ（現在歯数19本以下、20本以上）に分けられた。多変量ロジスティック回帰分析を使い、現在歯数19本以下であることに関連する要因を評価した。

**結果:**

対象者の現在歯数0本、1-9本、10-19本、20本以上の人数（%）はそれぞれ、1764(21.1%),1779(21.3%),1836(22.0%),2937(35.6%)であった。対象者全体と65-79歳では現在歯数19本以下であることは高齢、喫煙（現在喫煙、過去喫煙）、低い教育歴と有意な関連がみられた。また現在歯数19本以下であることは低BMI、低アルブミン値、低い現在飲酒者割合と有意な関連がみられた。40-64歳では、現在歯数19本以下であることは低HDL-C値と高HbA1c値と有意な関連がみられたが、65-79歳ではこの関連はみられなかった。

**結論:**

喫煙、低い教育歴、不良な栄養状態は日本人の中高年男性の歯牙喪失と関連していることが示唆された。

## INTRODUCTION

Tooth loss was found to be associated with systemic chronic diseases such as cardiovascular disease and cancer.^1^^,^^2^ In addition, tooth loss affects daily activities such as speaking, smiling, chewing, and tasting.^3^ Prevention of tooth loss thus helps maintain good general health and high quality of life.

Many studies in various countries have revealed factors related to tooth loss, including smoking,^4^^–^^6^ nutritional status,^7^^–^^11^ and educational level.^7^^,^^12^^–^^15^ In Japan, numerous studies have examined the association between smoking and tooth loss at all ages.^3^^,^^16^^–^^20^ There have also been several studies on the associations of tooth loss with nutritional status^21^^,^^22^ and educational level.^23^^,^^24^ However, because most of those studies were conducted among elderly populations, there is little information on factors related to tooth loss in middle-aged populations. Moreover, there have been few studies in Japan on the associations of tooth loss with laboratory variables.^25^

Thus, to identify factors associated with tooth loss among middle-aged and elderly men, we analyzed laboratory data and information on lifestyle and social factors from a large-scale community-dwelling Japanese population.

## METHODS

### Study population

We analyzed baseline data from the Iwate-Kenpoku cohort (Iwate-KENCO) study, which was designed as a cohort study of community-dwelling residents living in the north of the main island of Japan. The methodology of the Iwate-KENCO study has been described elsewhere.^26^ The baseline survey was carried out between 2002 and 2005. The original cohort members consisted of 26 469 participants aged 18 years or older who underwent annual health check-ups. Of the original cohort, we focused on 8476 men aged 40 to 79 years to identify factors associated with tooth loss. Among those 8476 male participants, we excluded 124 men with missing data on number of teeth. Ultimately, data from 8352 male participants (99% of the male participants aged 40–79 years) were used in the analysis. This study was approved by the Medical Ethics Committee of Iwate Medical University. All participants provided written informed consent.

### Measurements

Body mass index (BMI) was calculated as weight (kg) divided by the square of the height (m^2^). Systolic blood pressure (SBP) and diastolic blood pressure (DBP) were measured by trained nurses using an automatic device, with the participant in a sitting position after resting for at least 5 minutes. The average of 2 measurements was recorded. Casual blood samples were drawn from antecubital veins of seated participants. All samples were collected into vacuum tubes containing ethylenediaminetetraacetic acid (EDTA) or a serum separator gel. The samples were stored in an icebox immediately after collection, transported to a laboratory (Iwate Health Service Association), and analyzed on the same day. Serum levels of total cholesterol (TC) and high-density lipoprotein cholesterol (HDL-C) were measured by an enzymatic method. Measurements of TC and HDL-C have been standardized by the Osaka Medical Center for Health Science and Promotion—a member of the Cholesterol Reference Method Laboratory Network (CRMLN) controlled by the Centers for Disease Control and Prevention (Atlanta, GA, USA)^27^—and have met all criteria for both precision and accuracy of lipid measurement. Plasma glucose levels were determined by the hexokinase ultraviolet method. Glycosylated hemoglobin (HbA1c) was measured by high-performance liquid chromatography as a Japan Diabetes Society (JDS) value, which was then converted to a National Glycohemoglobin Standardization Program (NGSP) value by adding 0.4%. Serum albumin level was measured by the bromocresol green method. Serum level of high-sensitivity C-reactive protein (hsCRP) was determined by the latex-enhanced immunonephelometric method (Dade Behring Diagnostics, Germany). The detection limit of the hsCRP assay is 0.1 mg/dL, and hsCRP values under the minimum detectable level were recorded as 0.1 mg/L.

Each participant completed a self-administered questionnaire that included questions on medication, smoking habits, alcohol intake, years of education, marital status, and number of teeth. Number of teeth was assessed by a single question, “How many teeth do you have (0, 1–9, 10–19, or ≥20)?”. Diabetes was defined as a plasma glucose level of 200 mg/dL or higher, an HbA1c level (NGSP) of 6.5% or higher, or use of antidiabetic agents. Smoking status was classified into 3 categories: nonsmoking, ex-smoking, and current smoking. Alcohol drinking status was classified as current habitual drinking or no habitual drinking. Marital status was classified as married or single (including unmarried, divorced, and widowed). Low education level was defined as less than 10 years of education.

### Statistical analysis

We calculated age-adjusted means and age-adjusted proportions of variables in each group (ie, 0, 1–9, 10–19, ≥20 teeth), using analysis of covariance (ANCOVA) for continuous variables and logistic regression for categorical variables. Data for hsCRP were expressed as age-adjusted geometric means. Linear trend across the 4 groups was examined by linear regression for continuous variables and logistic regression for categorical variables. To determine factors associated with tooth loss, we classified participants into 2 groups according to number of teeth (≤19 teeth vs ≥20 teeth). Multivariate logistic regression was performed for all participants aged 40 to 79 years, with 19 or fewer teeth as the dependent variable (coded as 1 for having ≤19 teeth and 0 for having ≥20 teeth), and age, BMI, SBP, TC, HDL-C, albumin, log-transformed hsCRP, smoking status (current smoker, ex-smoker, or non-smoker), alcohol drinking status (current drinker or not), marital status (single or not), and education level (low or not) as independent variables. In addition, similar analyses were performed after stratification by age group (40–64 years vs 65–79 years). In all analyses, a 2-sided *P* value of less than 0.05 was considered to indicate statistical significance. The statistical package SPSS (version 11.0J) was used for the analysis.

## RESULTS

Among the 8352 men, the numbers (proportions) of men with 0, 1 to 9, 10 to 19, and 20 or more teeth were 1764 (21.1%), 1779 (21.3%), 1836 (22.0%), and 2973 (35.6%), respectively. The respective numbers (proportions) were 319 (8.8%), 570 (15.6%), 914 (25.1%), and 1840 (50.5%) among men aged 40 to 64 years and 1445 (30.7%), 1209 (25.7%), 922 (19.6%), and 1133 (24.1%) among men aged 65 to 79 years.

As shown in Table 1, mean age was higher in men with fewer teeth. After adjustment for age, number of teeth was positively associated with BMI, serum levels of TC, HDL-C, and albumin, and proportions of nonsmokers and current drinkers, and inversely associated with hsCRP and proportions of current smokers, single men, and men with low education level.

**Table 1.** Age-adjusted characteristics of men aged 40–79 years, by number of teeth

Number of teeth	0	1–9	10–19	≥20	*P* for trend
Number of participants (%)	1764 (21.1)	1779 (21.3)	1836 (22.0)	2973 (35.6)	
Age (years)	69.7 (6.3)	66.7 (7.8)	63.0 (9.4)	60.0 (9.9)	<0.001
BMI (kg/m^2^)	23.5 (23.3–23.6)	23.8 (23.7–24.0)	24.1 (24.0–24.3)	24.2 (24.1–24.3)	<0.001
SBP (mm Hg)	130.2 (129.4–131.1)	130.3 (129.4–131.2)	131.1 (130.2–132.0)	131.3 (131.1–132.0)	0.198
TC (mg/dL)	190.8 (189.2–192.3)	191.1 (189.6–192.6)	190.3 (188.8–191.8)	193.3 (192.1–194.5)	0.007
HDL-C (mg/dL)	55.6 (54.9–56.4)	56.3 (55.6–57.0)	55.2 (54.5–55.9)	56.7 (56.1–57.3)	0.006
HbA1c (%)	5.60 (5.56–5.63)	5.55 (5.52–5.59)	5.55 (5.51–5.58)	5.54 (5.51–5.57)	0.108
Albumin (g/dL)	4.40 (4.38–4.41)	4.38 (4.36–4.39)	4.39 (4.38–4.41)	4.42 (4.41–4.43)	<0.001
hsCRP (mg/L)	0.58 (0.55–0.61)	0.56 (0.53–0.59)	0.57 (0.54–0.60)	0.52 (0.50–0.54)	0.014
Diabetes (%)	10.8	11.0	11.0	10.0	0.658
Current smoker (%)	41.8	35.4	28.6	23.6	<0.001
Ex-smoker (%)	30.2	31.1	29.1	31.9	0.390
Nonsmoker (%)	28.2	32.3	40.3	42.9	<0.001
Current drinker (%)	55.0	61.1	64.1	65.1	<0.001
Single (%)	12.7	11.3	10.8	10.4	0.040
<10 years of education (%)	72.0	63.1	56.1	51.5	<0.001

Data are expressed as age-adjusted means (95% CIs) for continuous variables, except for age and hsCRP, and age-adjusted proportions for categorical variables. Data for age are expressed as means (SD), and data for hsCRP are expressed as age-adjusted geometric means (95% CI). Age-adjusted means and proportions were estimated, with the exception of missing data: the number of missing values was 11 for BMI, 2 for SBP, 2 for TC, 2 for HDL-C, 3 for HbA1c, 184 for hsCRP, 2374 for albumin, 286 for marital status, and 145 for years of education.

*P* values for linear trend tests across the 4 groups were estimated using linear regression for continuous variables and logistic regression for categorical variables. BMI, body mass index; SBP, systolic blood pressure; TC, total cholesterol; HDL-C, high-density lipoprotein cholesterol; HbA1c, glycosylated hemoglobin; hsCRP, high-sensitivity C-reactive protein.

Table 2 shows adjusted odd ratios (ORs) and their 95% CIs for having 19 or fewer teeth among men aged 40 to 79 years. Older men, current smokers, and men with a low education level had a significantly higher risk of having 19 or fewer teeth. In contrast, men with high BMI, men with high serum albumin, and current drinkers had a significantly lower risk of having 19 or fewer teeth. Among men aged 40 to 64 years (Table 3), older men, men with a high HbA1c level, current smokers, and men with a low education level had a significantly higher risk of having 19 or fewer teeth, whereas men with a high BMI and high HDL-C had a significantly lower risk of having 19 or fewer teeth. Among men aged 65 to 79 years (Table 4), older men, current smokers, ex-smokers, and men with a low education level had a significantly higher risk of having 19 or fewer teeth, whereas men with a high BMI, men with a high albumin level, and current drinkers had a significantly lower risk of having 19 or fewer teeth.

**Table 2.** Adjusted odds ratios (ORs) and 95% CIs for having ≤19 teeth among men aged 40–79 years

		OR	95% CI	*P* value
Age	(per 10-year increase)	2.05	(1.90–2.21)	<0.001
BMI	(per 1-kg/m^2^ increase)	0.96	(0.94–0.99)	0.001
SBP	(per 10-mm Hg increase)	1.00	(0.97–1.03)	0.938
TC	(per 10-mg/dL increase)	1.00	(0.98–1.02)	0.852
HDL-C	(per 10-mg/dL increase)	0.97	(0.93–1.01)	0.144
HbA1c	(per 1% increase)	1.01	(0.94–1.09)	0.774
Albumin	(per 1-g/dL increase)	0.70	(0.55–0.88)	0.002
hsCRP	(per log(1)-mg/L increase)	1.05	(0.99–1.11)	0.084
Current smoker	(vs. nonsmoker)	1.66	(1.42–1.93)	<0.001
Ex-smoker	(vs. nonsmoker)	1.19	(1.03–1.37)	0.019
Current drinker	(vs. other)	0.77	(0.68–0.88)	0.000
Single	(vs. marriage)	0.97	(0.81–1.17)	0.748
<10 years of education	(vs. ≥10 yrs)	1.57	(1.39–1.78)	<0.001

Logistic regression analysis was performed using data from 5706 participants with complete data.

BMI, body mass index; SBP, systolic blood pressure; TC, total cholesterol; HDL-C, high-density lipoprotein cholesterol; HbA1c, glycosylated hemoglobin; hsCRP, high-sensitivity C-reactive protein.

**Table 3.** Adjusted odds ratios (ORs) and 95% CIs for having ≤19 teeth among men aged 40–64 years

		OR	95% CI	*P* value
Age	(per 10-year increase)	1.82	(1.58–2.09)	<0.001
BMI	(per 1-kg/m^2^ increase)	0.96	(0.93–0.99)	0.012
SBP	(per 10-mm Hg increase)	1.02	(0.97–1.07)	0.445
TC	(per 10-mg/dL increase)	1.00	(0.98–1.03)	0.879
HDL-C	(per 10-mg/dL increase)	0.94	(0.88–1.00)	0.042
HbA1c	(per 1% increase)	1.13	(1.01–1.27)	0.030
Albumin	(per 1-g/dL increase)	0.78	(0.56–1.07)	0.128
hsCRP	(per log(1)-mg/L increase)	1.07	(0.98–1.16)	0.114
Current smoker	(vs. nonsmoker)	1.42	(1.16–1.73)	0.001
Ex-smoker	(vs. nonsmoker)	1.04	(0.84–1.30)	0.706
Current drinker	(vs. other)	0.93	(0.77–1.12)	0.443
Single	(vs. marriage)	1.00	(0.78–1.27)	0.978
<10 years of education	(vs. ≥10 yrs)	1.52	(1.29–1.81)	<0.001

Logistic regression analysis was performed using data from 2523 participants with complete data.

BMI, body mass index; SBP, systolic blood pressure; TC, total cholesterol; HDL-C, high-density lipoprotein cholesterol; HbA1c, glycosylated hemoglobin; hsCRP, high-sensitivity C-reactive protein.

**Table 4.** Adjusted odds ratios (ORs) and 95% CIs for having ≤19 teeth among men aged 65–79 years

		OR	95% CI	*P* value
Age	(per 10-year increase)	2.53	(2.01–3.18)	<0.001
BMI	(per 1-kg/m^2^ increase)	0.97	(0.94–1.00)	0.039
SBP	(per 10-mm Hg increase)	0.98	(0.94–1.03)	0.385
TC	(per 10-mg/dL increase)	0.99	(0.96–1.02)	0.602
HDL-C	(per 10-mg/dL increase)	1.00	(0.94–1.06)	0.995
HbA1c	(per 1% increase)	0.92	(0.83–1.02)	0.116
Albumin	(per 1-g/dL increase)	0.63	(0.45–0.87)	0.006
hsCRP	(per log(1)-mg/L increase)	1.03	(0.95–1.12)	0.439
Current smoker	(vs. nonsmoker)	1.91	(1.51–2.42)	<0.001
Ex-smoker	(vs. nonsmoker)	1.28	(1.05–1.55)	0.014
Current drinker	(vs. other)	0.66	(0.55–0.80)	<0.001
Single	(vs. marriage)	0.84	(0.63–1.13)	0.245
<10 years of education	(vs. ≥10 yrs)	1.68	(1.40–2.02)	<0.001

Logistic regression analysis was performed using data from 3138 participants with complete data.

BMI, body mass index; SBP, systolic blood pressure; TC, total cholesterol; HDL-C, high-density lipoprotein cholesterol; HbA1c, glycosylated hemoglobin; hsCRP, high-sensitivity C-reactive protein.

## DISCUSSION

In this study of 8532 community-dwelling men aged 40 to 79 years, having 19 or fewer teeth was significantly associated with older age, smoking status (current smoking and ex-smoking), and low education level. In addition, men with 19 or fewer teeth were more likely to have a low BMI and low serum albumin level and less likely to be current alcohol drinkers. In addition, among men aged 40 to 64 years, men with 19 or fewer teeth were more likely to have a low serum HDL-C level and a high HbA1c level.

Studies have shown that tooth loss is associated with smoking in middle-aged and elderly populations,^4^^–^^6^ as was observed in the present study. In Japan, a nationwide study of 3999 adults aged 40 years or older showed that, as compared with nonsmokers, current smokers had a significant 2.22-fold risk of having 19 or fewer teeth and that the difference in risk for ex-smokers was not significant.^18^ A retrospective cohort study of 547 men aged 55 to 75 years showed that current smokers and ex-smokers had significant 1.96-fold and 1.86-fold risks of having more than 8 missing teeth, respectively.^16^ That study also showed that ex-smokers who had smoked for 21 years or longer had a significantly higher risk of having more than 8 missing teeth, as compared with never smokers, and that the risk of having more than 8 missing teeth among ex-smokers who had stopped smoking for more than 21 years was equal to that of never smokers.^16^ The present study showed that, as compared with never smokers, male ex-smokers aged 65 to 79 years, but not those aged 40 to 64 years, had a significantly higher risk of having 19 or fewer teeth. Mean number of smoking years was higher in men aged 65 to 79 years (29.3 years) than in men aged 40 to 64 years (20.8 years). The longer duration of smoking among elderly men may increase the risk of having 19 or fewer teeth among ex-smokers.

In this study, low education level was significantly associated with tooth loss among men aged 40 to 64 years and men aged 65 to 79 years. This finding is consistent with the results of previous studies in various populations.^7^^,^^12^^–^^15^ Low education level was found to be associated with less utilization of dental care^28^ and unfavorable oral health-related behaviors,^29^ which could lead to tooth loss. In Japan, a study of 1201 community residents aged 55 to 75 years showed that those with higher educational levels had a greater likelihood of having 20 or more teeth.^23^ Another study, of elderly Japanese aged 65 years or older, showed that subjects with 9 years of education or less had a significantly higher risk of having 19 or fewer teeth than did those with at least 13 years of education.^24^ These present and past findings suggest a significant association between low education level and tooth loss among both the elderly and middle-aged populations in Japan.

We found that having 19 or fewer teeth was significantly associated with low BMI (among the participants overall) and with low serum albumin (among men aged 65–79 years). These findings are consistent with the results of previous studies^7^^,^^8^^,^^11^ and suggest that people with fewer teeth, particularly elderly adults, may have poor nutritional status. People with fewer teeth may also have impaired masticatory function that limits their dietary choices and affects their nutritional status.^10^ A study of 6985 US adults found that those with fewer than 28 teeth had significantly lower intakes of carrots, tossed salads, and dietary fiber and lower serum levels of beta carotene, folate, and vitamin C than did fully dentate adults.^9^ In Japan, a study of 20 366 dentists showed a decreasing trend in intakes of carotene and vitamins A and C with increasing number of teeth lost. That study also showed similar trends for consumption of milk and dairy products and green-yellow vegetables, whereas consumption of rice and confectioneries was inversely associated with number of remaining teeth.^21^

In this study, having 19 or fewer teeth was associated with low serum HDL-C and high HbA1c among men aged 40 to 64 years but not among those aged 65 to 79 years. Diabetes is a major risk factor for periodontal disease,^30^ and periodontal disease was found to be associated with the development of glucose intolerance.^31^ In addition, several studies found that people with periodontal disease have low HDL-C levels.^32^^,^^33^ Therefore, the significant associations of tooth loss with HDL-C and HbA1c among the middle-aged men in this study may reflect associations of periodontal disease with HDL-C and HbA1c, although we had no information on the causes of tooth loss. In contrast, as mentioned above, tooth loss in elderly adults may reflect poor nutritional status, expressed as low BMI and low albumin level, rather than the presence of periodontal disease.

There were several limitations in this study. First, the cross-sectional design of the present study does not allow identification of casual relationships. Second, number of teeth was assessed by a self-administered questionnaire, without clinical examination. However, self-reported number of teeth was found to be highly correlated with actual number of teeth at a clinical examination in a general population.^34^ Third, our subjects were middle-aged and elderly Japanese men living in the north of the main island of Japan, which limits the generalizability of the present results.

In conclusion, we found that smoking, low education level, and poor nutritional status were associated with tooth loss among middle-aged and elderly men. These risk indicators should be considered when planning oral health programs for both elderly and middle-aged adults.

## ONLINE ONLY MATERIALS

Abstract in Japanese.
