# Supplementary material for: Factors Related to Tooth Loss Among Community-Dwelling Middle-aged and Elderly Japanese Men
Source: J Epidemiol. 2013 Jul 5;23(4):301–6. doi: 10.2188/jea.JE20120180 (PMC3709550; doi:10.2188/jea.JE20120180)
Supplement: Abstract in Japanese. [file je-23-301-s001.pdf]

地域住民における中高年男性の歯牙喪失に関連する要因

安藤歩[1], 大澤正樹[1], 八重樫由美[1], 坂田清美[1], 丹野高三[1], 小野田敏行[1], 板井一好[2], 田中文隆[3], 蒔田真司[3], 大間々真一[4], 小笠原邦昭[4], 小川彰[4], 石橋靖宏[5], 栗林徹[6], 小山富子[7], 岡山明[2]

1 岩手医科大学医学部衛生学公衆衛生学講座
2 結核予防会第一健康相談所生活習慣病予防研究センター
3 岩手医科大学医学部内科学講座心血管・腎・内分泌内科分野
4 岩手医科大学医学部脳神経外科学講座
5 岩手医科大学医学部内科学講座神経内科・老年科分野
6 岩手大学教育学部
7 岩手県予防医学協会

抄録

背景目的:大規模な日本人の地域住民のデータを使用し、中高年男性の歯牙喪失に関連する要因を明らかにすること。

方法: 2002 年-2005 年に市町村による基本健診を受診し、研究参加に同意した東北地域（岩手県北部地域）に住む 40-79 歳の男性 8352 名を対象とした。現在歯数は単一の質問（あなたは何本の歯を持っていますか？（0 本、1-9 本、10-19 本、20 本以上））によって評価された。対象者はその質問の答えによって 2 つのグループ（現在歯数 19 本以下、20 本以上）に分けられた。多変量ロジスティック回帰分析を使い、現在歯数 19 本以下であることに関連する要因を評価した。

結果: 対象者の現在歯数 0 本、1-9 本、10-19 本、20 本以上の人数（%）はそれぞれ、1764(21.1%),1779(21.3%),1836(22.0%),2937(35.6%)であった。対象者全体と 65-79 歳では現在歯数 19 本以下であることは高齢、喫煙（現在喫煙、過去喫煙）、低い教育歴と有意な関連がみられた。また現在歯数 19 本以下であることは低 BMI、低アルブミン値、低い現在飲酒者割合と有意な関連がみられた。40-64 歳では、現在歯数 19 本以下であることは低 HDL-C 値と高 HbA1c 値と有意な関連がみられたが、65-79 歳ではこの関連はみられなかった。

結論: 喫煙、低い教育歴、不良な栄養状態は日本人の中高年男性の歯牙喪失と関連していることが示唆された。

キーワード: 歯牙喪失、リスク指標、中年男性、高齢者男性、日本人、横断研究
